# Disparities in the consensus for treatment of chemotherapy-induced thrombocytopenia

**DOI:** 10.3332/ecancer.2023.1627

**Published:** 2023-11-13

**Authors:** Liana Hambardzumyan, Henrik Grigoryan, Maria Badikyan, Heghine Khachatryan, Nelly Sargsyan, Arliette Sulikhanyan, Gevorg Tamamyan, Justin Stebbing

**Affiliations:** 1 Hematology Center after Prof. R. H. Yeolyan, Yerevan 0014, Armenia; 2 Department of Surgery and Cancer, Imperial College, London SW7 2BX, UK; 3 Department of Pediatric Oncology and Hematology, Yerevan State Medical University, Yerevan 0025, Armenia; 4 Pediatric Cancer and Blood Disorders Center of Armenia, Hematology Center after Prof. R. H. Yeolyan, Yerevan 0014, Armenia; 5 Immune Oncology Research Institute, Yerevan 0014, Armenia; 6 California Department of Public Health, Los Angeles, CA 95899-7377, USA

**Keywords:** platelets, thrombocytopenia, guidelines, chemotherapy

## Abstract

**Introduction:**

Chemotherapy-induced thrombocytopenia (CIT) is an arduous complication of chemotherapy to be dealt with, and there are many unmet needs in this field to be addressed on the global front. We have conducted this study to contribute to the understanding of existing knowledge gaps of CIT management and highlight the direction to focus future investigations.

**Methods:**

This was an academic single-institution report on a cross-sectional study evaluating CIT management practices using platelet (PLT) transfusions by haematologists and oncologists in Armenia.

**Results:**

Physicians’ opinions differed significantly when it came to defining thrombocytopenia by PLT levels. 13.2% of those surveyed considered thrombocytopenia to be when PLT counts fall below 180 × 10^9^/L, 42.1% defined thrombocytopenia to have a PLT threshold of 150 × 10^9^/L, 15.8% and 21.0% specialists setting their thresholds at 140 × 10^9^/L and 100 × 10^9^/L, respectively.

All physicians managed CIT by performing PLT transfusions for prophylactic purposes (i.e., when PLT count falls below a certain threshold) with none of them transfusing PLTs only on-demand to address active bleeding. 73.3% haematologists (adult), 57.1% medical oncologists, and 50% paediatricians deemed 10 × 10^9^/L as the threshold PLT count for transfusing afebrile patients with haematologic malignancies (besides acute promyelocytic leukaemia (APL)) and solid tumours.

PLT products availability varied among the respondents, with only 53% of them responding that they had 24/7 access.

**Conclusion:**

CIT is a complication of interest to physicians worldwide and has not been resolved yet. This is the first conducted survey regarding CIT and the initial step for further research.

## Introduction

**Figure 1.**
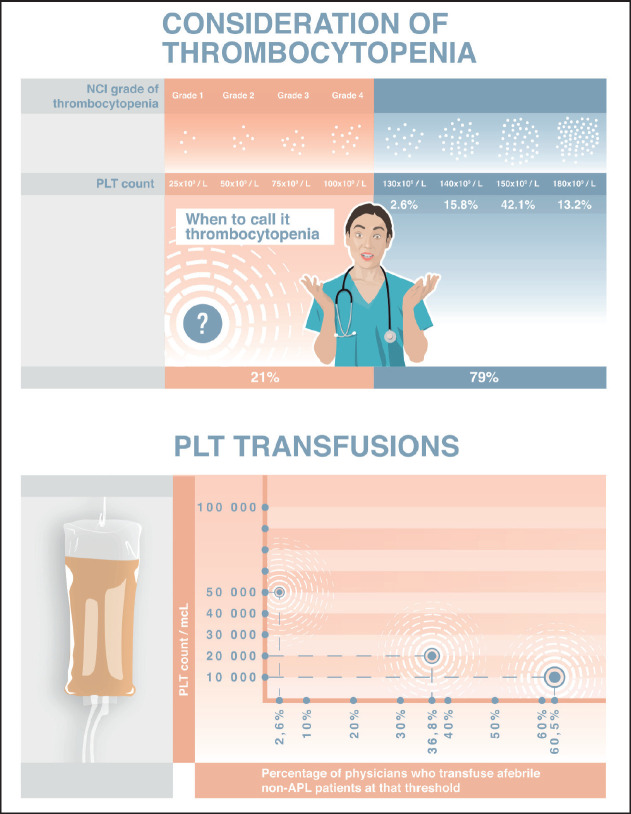
(a): According to NCI criteria there are 4 grades of thrombocytopenia based on PLT thresholds: 75 to <100 × 10^9^/L (Grade 1), 50 to <75 × 10^9^/L (Grade 2), 25 to <50 × 10^9^/L (Grade 3); <25 × 10^9^/L (Grade 4). However, only 21.0% of our respondents defined thrombocytopenia to have a PLT threshold of 100,000/mcL, while the majority (42.1%) denoted a threshold of 150,000/mcL. Also 15.8% and 13.2% of those surveyed considered thrombocytopenia to be when PLT counts fall below 140,000/mcL and 180,000/mcL, respectively. A threshold of 130,000/mcL was mentioned by 2.6% of the specialists. (b): All physicians preferred PLT transfusions for prophylactic purposes (not only on demand). Afebrile patients (non-APL) would be transfused when having PLT count below 10,000/mcL by 60.5% of physicians whereas 36.8% and 2.6% of specialists performed transfusions when PLT counts fell below 20,000/mcL and 50,000/mcL, respectively.

Chemotherapy-induced thrombocytopenia (CIT) usually refers to the decrease of platelet (PLT) counts in peripheral blood below 100 × 10^9^/L for 3–4 weeks following the last chemotherapy, which may result in chemotherapy delays and/or dose reductions [1]. It is a frequent haematologic complication of myelosuppressive cancer therapy where incidence and prevalence vary greatly by cancer type and chemotherapy regimen. A review of published studies shows that CIT occurs in about 10%–40% of patients with solid tumours and 40%–70% of patients with haematologic malignancies [2–4]. To be considered clinically significant, PLT counts must fall below 50 × 10^9^/L (grades 3 and 4 thrombocytopenia (The National Cancer Institute (NCI), common terminology criteria for adverse events, (version 5.0), shown in Figure 1a). This affects approximately 4% (grade 3) and 2% (grade 4) of patients with solid tumours and 16% and 12%, respectively, of patients with haematologic malignancies [4]. The highest prevalence of thrombocytopenia among solid tumour patients was observed in these cancer types in descending order: colorectal, non-small cell lung, and ovarian [5]. The main mechanism of thrombocytopenia is myelosuppression, but immune-mediated mechanisms, splenic sequestration and other issues also directly impact PLT release. Molecular mechanisms include inhibition of platelet-derived growth factor (PDGF), and apoptosis of megakaryocytes, or release of toxic mediators into the bone marrow milieu which may also play a role [6–9].

Currently, there are no standardized guidelines nor Food and Drug Administration-approved agent for the prevention or treatment of CIT. According to published literature, thrombopoietin receptor agonists (TPO-RA) improve PLT count in the majority of cases. Consistent maintenance of TPO-RA may allow for the resumption of chemotherapy without recurrence of CIT but it is not included in the guidelines for CIT management yet [2, 10, 11]. PLT transfusions can provide only temporary improvement and are not a rational treatment option during chemotherapy cycles for an extended period. Regardless, they are still the main treatment approach for CIT, including resource-poor settings. Chemotherapy dosage, cycle reduction and/or treatment delays also play a role in CIT management, reducing bleeding risk and the need for frequent PLT transfusions. These may, however, result in the reduction of relative dose intensity – a consequence that may impact the progression-free and overall survival [12–14].

CIT is considered an arduous complication of chemotherapy to be dealt with, and there are many unmet needs in this field to be addressed on the global front. We have conducted this study to contribute to the understanding of existing knowledge gaps of CIT management and highlight the direction to focus future investigations.

## Materials and methods

This was an academic single-institution report on a cross-sectional study evaluating CIT management practices using PLT transfusions by haematologists and oncologists in Armenia. This study was conducted at the only haematology centre in Armenia, where adult and paediatric patients with haematological malignancies and paediatric cancer (both solid and liquid tumours) patients are managed. The centre also has a medical oncology department providing cancer care to adult patients with solid tumours.

The survey was conducted among all haematologists, oncologists and clinical residents providing care at departments of adult haematology (out- and inpatients), Pediatric Cancer and Blood Disorders Center of Armenia (situated at the Hematology Center), and the medical oncology department. The participants were asked to complete our self-administered cross-sectional survey and were informed that they could stop the survey at any moment or skip any questions they deemed to be uncomfortable (Appendix).

Questions used to generate the data for this study were organized into four sections. The first section enquired about their specialization and years of experience. The second section included questions assessing the evaluation of thrombocytopenia. The third section aimed to evaluate management approaches of CIT (depending on their specializations) by PLT transfusions, as well as PLT count thresholds for febrile and afebrile patients of various cancer types. The fourth section was dedicated to deducing accessibility of PLT products and details regarding transfusion costs.

## Results

In total, 38 physicians completed the survey: 15 (39.5%) of them were haematologists (adult), 7 (18.4%) were medical oncologists, 6 (15.8%) paediatric haematologists, 3 (7.9%) paediatric oncologists and 7 (18.4%) were paediatric oncology/haematology residents. Twenty-five (65.8%) of the physicians had less than 6 years of professional experience, 6 (15.8%) physicians had more than 10 years and 7 (18.4%) physicians had more than 20 years.

Physicians’ opinions differed significantly when it came to defining thrombocytopenia by PLT levels. Five (13.2%) of those surveyed considered thrombocytopenia to be when PLT counts fall below 180 × 10^9^/L. Of these, it was notable that 4 of the 5 had less than 6 years of professional experience, with one having 11 years. In contrast, 16 (42.1%) of those surveyed, defined thrombocytopenia to have a PLT threshold of 150 × 10^9^/L, with another six (15.8%) and eight (21.0%) specialists setting their thresholds at 140 × 10^9^/L and 100 × 10^9^/L, respectively. One physician denoted a threshold of 130 × 10^9^/L, and two participants did not provide an actual number – instead, noting that they consider thrombocytopenia when PLT counts fall below the ‘normal᾿ level (Figure 1a).

All 38 physicians managed CIT by performing PLT transfusions for prophylactic purposes (i.e., when PLT count falls below a certain threshold) with none of them transfusing PLTs only on-demand to address active bleeding. Eleven (73.3%) haematologists (adult), four (57.1%) medical oncologists, and eight (50%) paediatricians deemed 10 × 10^9^/L as the threshold PLT count for transfusing afebrile patients with haematologic malignancies (besides acute promyelocytic leukaemia (APL)) and solid tumours. Only one paediatrician preferred to transfuse when PLTs were less than 50 × 10^9^/L. The other 14 (36.8%) of the 38 respondents used a threshold PLT count of 20 × 10^9^/L (Figure 1b).

Febrile patients (non-APL) would be transfused when having PLT count below 20 × 10^9^/L by 13 (81.3%) haematologists (adult), four (57.1%) medical oncologists, and 11 (73.3%) paediatricians. One haematologist (adult) and two paediatricians preferred to transfuse febrile patients when PLT count was below 30 × 10^9^/L, whereas three medical oncologists and one paediatrician transfused when PLT count was less than 10 × 10^9^/L. Three participants identified less than 50,000/mcL as their threshold; two of these individuals had less than 6 years of professional experience, and one individual had more than 30 years.

Afebrile and febrile adult APL patients would receive transfusions according to 12 (75%) and 11 (73.3%) haematologists (adult) when they have PLT count below 20 × 10^9^/L, respectively. Another three (25%) and four (26.7%) haematologists (adult) consider a threshold PLT count less than 50 × 10^9^/L to transfuse PLTs to afebrile and febrile APL patients. Nine (56.3%) paediatricians would transfuse both afebrile and febrile patients with APL when PLT falls below 50 × 10^9^/L, with one specialist transfusing when PLT was less than 10 × 10^9^/L for both groups. Three paediatricians denoted a threshold of 20 × 10^9^/L as the transfusion threshold for afebrile patients, with one paediatrician noting that threshold as acceptable for febrile patients. Another two paediatricians set a transfusion threshold of 75 × 10^9^/L for febrile APL patients.

PLT products availability varied among the respondents, with only 20 (53%) of them responding that they had 24/7 access.

67% of haematologists (adult) and medical oncologists mentioned that the cost of PLT products affects their decision on making a prophylactic transfusion as adult patients have to pay for these. Paediatricians, on the other hand, did not have that issue as charity foundations covered the cost of complete management for children with cancer, including providing needed blood products [15].

## Discussion

Numerous clinical questions are raised when it comes to decision making such as whether to transfuse PLT prophylactically or therapeutically and which PLT count to use as a threshold for prophylactic transfusion. This especially applies to a low-income country such as ours, where drugs are often inaccessible. A review of randomized clinical trials reports that a therapeutic-only strategy is associated with an increased risk of low-to moderate-grade bleeding and reduced number of transfusions per patient. However, these associations occurred in patients with haematologic malignancies who had anamnesis of myelosuppressive therapy or haematopoietic stem cell transplantation (HSCT) [16, 17]. According to the 2017 American Society for Clinical Oncology guidelines prophylactic PLT transfusions are recommended if the bone marrow is suppressed (including cytotoxic chemotherapy) when PLT count falls below a certain threshold. The threshold varies according to the patient’s diagnosis, clinical condition, and treatment modality. A PLT count of 10 × 10^9^/L is generally used as the threshold in patients receiving treatment for haematologic malignancies who are hospitalized, afebrile, and without active bleeding or infection. APL is considered an exception because of higher bleeding risk, so it is recommended to transfuse PLTs when the PLT count is below 30 × 10^9^/L and up to 50 × 10^9^/L. Higher thresholds may be needed under the following conditions: if fever, sepsis or coagulopathy is present, or if the patient is not hospitalized and/or cannot be closely monitored [18]. Randomized trials of PLT transfusion threshold in patients with solid tumours have not been performed but observational studies support 10 × 10^9^/L as a threshold. For necrotic tumours, 20 × 10^9^/L may be appropriate due to a higher risk of active bleeding or the need for invasive procedure [18].

One must take into account the costs and benefits of PLT transfusions because repeated transfusions can increase the risk of an array of potential adverse health effects [19]. These events can be immune-mediated such as febrile non-haemolytic transfusion reaction (FNHTR), allergic/anaphylaxis, TAGvHD, transfusion-related acute lung injury (TRALI), post-transfusion purpura, transfusion-related immunomodulation, and PLT refractoriness [19]. They can also be non-immune mediated events such as transfusion-associated circulatory overload, physical injury, sepsis, viral infection transmission and hypotensive reaction [19]. Although the majority of the aforementioned events are not dangerous, they are quite common. For example, febrile FNHTR and ‘allergic’ reactions are observed in up to 20%–30% of cases [20, 21]. According to Canadian health statistics, one in 50,000 PLT transfusions is associated with bacterial contamination, one in 153,000 with hepatitis B virus (HBV) infection, one in 5,000 can cause TRALI [22]. In contrast, 1 in ten PLT transfusions may cause febrile reactions [22]. Not only do increased transfusions result in a considerable potential risk to patients (transfusions reactions, infection and alloimmunization), but may also be associated with increased cancer-related mortality in adult studies [23–25].

Over the past decade, the number of PLT units issued in the United Kingdom by the NHS Blood and Transplant (NHSBT) service to hospitals has steadily increased; it has risen by just over 25% in the 9 years prior to April of 2016 [26]. PLTs are the most expensive commonly used component supplied by NHSBT, with one unit costing £240.90 (both apheresis and pooled, 1 ATD) [27]. This price has increased since 2016 when it was valued at £193.15. Additionally, there are added value service costs that must be accounted for (irradiation, etc.) [27]. There is evidence that the inappropriate use of PLTs is an ongoing problem [28, 29]. To illustrate this, it is estimated that, 28% of PLT transfusions could have been avoided in 2010 [30]. Moreover, according to the US National Blood Collection and Utilization Survey report, about 1.3 billion USD was spent for PLT transfusions in the USA only in 2008 and about 2/3 of those transfusions were performed for prophylactic purposes without enough proof of their clinical benefit [31].

The seriousness of CIT consequences is variable ranging from less significant issues such as petechiae and ecchymosis, all the way up to life-threatening bleeding [32]. Although the main danger of thrombocytopenia is associated with morbidity and mortality due to excessive bleeding, other potential costs that must be considered are side effects due to PLT transfusions, or decreased efficacy of treatment due to delays, dosage and cycle reductions in therapy [33–35].

## Limitations

Several limitations of the current study must be noted. This was a questionnaire study conducted on a small number of physicians and clinical residents who were from the same region, thus reducing the generalizability of the findings, without a validation cohort elsewhere. However, the response rate was 100% eliminating certain biases and additionally, the questionnaire was pilot-tested. Barriers were measured using only a few questions, thus limiting our ability to generalize to a wide variety of issues. To address this, future studies should attempt to explore various realms of health and healthcare delivery by using more comprehensive measures. Despite these limitations, this study may serve as an initial step for further research, and for the introduction of several guidelines.

## Conclusion

Although our centre is the only specialized haematology centre in Armenia, consensus for CIT management, as well as the definition and concept of thrombocytopenia, varies. There are also no accepted and definite guidelines for either. There is ample work to be done to address the knowledge gap regarding thrombocytopenia, and its management among our specialists. Compounding this, there is a need for advocacy and implementation of appropriate guidelines such that solutions can be found for this outstanding issue. Furthermore, access to PLT products is an important barrier to address as only half of the surveyed physicians responded having 24/7 access to such resources. The health system may benefit from further investigations into such discordance of resource-use, and the employment of strategies to reduce the number of inappropriate blood requests and transfusions.

CIT is a complication of interest to physicians worldwide and has not been resolved yet. CIT requires a global approach to whole system implementation such as social support networks and cooperation of specialists on the international scale. This is the first survey conducted regarding CIT and the initial step for further research, as we are going to do a larger survey soon for the upcoming Delphi study.

## Declarations

The permission to access and use the data was received from the local board of Committee of Ethics of the Hematology Center after Prof. R. H. Yeolyan. Consent was obtained from all participants prior to data collection. All the selected respondents were given assurance of confidentiality that the information gathered will be used exclusively for research purposes.

## Conflicts of interest

Gevorg Tamamyan declares a research grant from Agenus Inc. and an advisory role at the Luzsana Biotechnology.

JS conflicts are listed here and none are relevant: https://www.nature.com/onc/editors

## Funding

No funding was received for the current work.

## Author contributions

LH: Conceptualization, methodology, validation, formal analysis, data curation, writing – original draft, writing – review and editing. HG: Conceptualization, validation, formal analysis, data curation, writing – original draft, writing – review and editing. MB: Validation, formal analysis, writing – original draft, writing – review and editing. HK: Validation, resources, writing – review and editing, supervision. NS: Validation, resources, writing – original draft, writing – review and editing. AS: Writing – reviewing and editing, writing – review and editing. JS: Supervision, validation, writing – review and editing. GT: Conceptualization, methodology, resources, writing – review and editing, supervision, validation.

## Availability of data and materials

The data underlying this article are available in the article and in its accompanying questionnaires in the Appendix.

## Appendix. Survey questionnaires ‘CIT and transfusion of PLT components’.

For adult haematology specialists

1. Are you a:

• Haematologist

• Haematology resident

2. How long have you worked with blood disorders?

______________________ year (s)

3. When do you consider thrombocytopenia?

PLT level below ______________________

4. When do you perform PLT transfusions in patients >18 years of age with haematologic malignancies (except acute APL) ?

a) For prophylaxis, when PLT count is below from 75,000/mcL

• from 50,000/mcL

• from 20,000/mcL

• from 10,000/mcL

• from 5,000/mcL

• Other ______________________

b) Only on-demand (in case of bleeding)

• Minor (petechiae, epistaxis) bleeding

• Heavy bleeding

• In both of the above-mentioned cases

5. When do you perform PLT transfusions in patients >18 years of age with APL?

a) For prophylaxis, when PLT count is below

• from 75,000/mcL

• from 50,000/mcL

• from 20,000/ mcL

• from 10,000/mcL

• from 5,000/mcL

• Other ______________________

b) Only on-demand (in case of bleeding)

• Minor (petechiae, epistaxis) bleeding

• Heavy bleeding

• In both of the above-mentioned cases

6. When do you perform PLT transfusions in febrile patients >18 years of age with haematologic malignancies (except APL)?

a) For prophylaxis, when PLT count is below

• from 75,000/mcL

• from 50,000/mcL

• from 20,000/ mcL

• from 10,000/mcL

• from 5,000/mcL

• Other ______________________

b) Only on-demand (in case of bleeding)

• Minor (petechiae, epistaxis) bleeding

• Heavy bleeding

• In both of the above-mentioned cases

7. When do you perform PLT transfusions in febrile patients >18 years of age with APL?

a) For prophylaxis, when PLT count is below

• from 75,000/mcL

• from 50,000/mcL

• from 20,000/mcL

• from 10,000/mcL

• from 5,000/mcL

• Other ______________________

b) Only on-demand (in case of bleeding)

• Minor (petechiae, epistaxis) bleeding

• Heavy bleeding

• In both of the above-mentioned cases

8. Is there round-the-clock availability of PLT components (if not, how do you manage thrombocytopenia, and what measures do you take in case of unavailability?

• Yes

• No ______________________

9. Do patients pay for PLT components out-of-pocket?

• Yes

• No

10. Does your selection of the PLT component (i.e., pooled or apheresis PLTs ) depend on the difficulty of the patient's solvency?

• Yes

• No

• Other ______________________

For adult oncology specialists

1. Are you an:

• Oncologist

• Oncology resident

2. How long have you worked with solid tumour patients?

______________________ year (s)

**3. **When do you consider thrombocytopenia?

PLT level below ______________________

4. When do you perform PLT transfusions in patients with solid tumours >18 years of age?

a) For prophylaxis, when PLT count is below

• from 75,000/mcL

• from 50,000/mcL

• from 20,000/mcL

• from 10,000/mcL

• from 5,000/mcL

• Other ______________________

a) Only on-demand (in case of bleeding)

• Minor (petechiae, epistaxis) bleeding

• Heavy bleeding

• In both of the above-mentioned cases

5. When do you perform PLT transfusions in febrile patients with solid tumours >18 years of age?

a) For prophylaxis, when PLT count is below

• from 75,000/mcL

• from 50,000/mcL

• from 20,000/mcL

• from 10,000/mcL

• from 5,000/mcL

• Other _______________

b) Only on-demand (in case of bleeding)

• Minor (petechiae, epistaxis) bleeding

• Heavy bleeding

• In both of the above-mentioned cases

6. Is there round-the-clock availability of PLT components (if not, how do you manage thrombocytopenia, and what measures do you take in case of unavailability?

• Yes

• No ______________________

7. Do patients pay for PLT components out-of-pocket?

• Yes

• No

8. Does your selection of the PLT component (i.e., pooled or apheresis PLTs) depend on the difficulty of the patient's solvency?

• Yes

• No

• Other ________________

For paediatric haematology specialists

1. How long have you worked with blood disorders among patients under 18 years of age? _______ year (s)

2. When do you consider thrombocytopenia?

PLT level below __________________

3. When do you perform PLT transfusions in patients <18 years of age with haematologic malignancies (except APL)?

a) For prophylaxis, when PLT count is below

• from 75,000/mcL

• from 50,000/mcL

• from 20,000/mcL

• from 10,000/mcL

• from 5,000/mcL

• Other ______________________

b) Only on-demand (in case of bleeding)

• Minor (petechiae, epistaxis) bleeding

• Heavy bleeding

vIn both of the above-mentioned cases

4. When do you perform PLT transfusions in patients <18 years of age with APL?

a) For prophylaxis, when PLT count is below

• from 75,000/mcL

• from 50,000/mcL

• from 20,000/mcL

• from 10,000/mcL

• from 5,000/mcL

• Other ______________________

b) Only on-demand (in case of bleeding)

• Minor (petechiae, epistaxis) bleeding

• Heavy bleeding

• In both of the above-mentioned cases

5. When do you perform PLT transfusions in febrile patients <18 years of age with haematologic malignancies (except APL)?

a) For prophylaxis, when PLT count is below

• from 75,000/mcL

• from 50,000/mcL

• from 20,000/mcL

• from 10,000/mcL

• from 5,000/mcL

• Other ______________________

b) Only on-demand (in case of bleeding)

• Minor ( petechiae, epistaxis) bleeding

• Heavy bleeding

• In both of the above-mentioned cases

6. When do you perform PLT transfusions in febrile patients <18 years of age with APL?

a) For prophylaxis, when PLT count is below

• from 75,000/mcL

• from 50,000/mcL

• from 20,000/mcL

• from 10,000/mcL

• from 5,000/mcL

• Other ______________________

b) Only on-demand (in case of bleeding)

• Minor ( petechiae, epistaxis) bleeding

• Heavy bleeding

• In both of the above-mentioned cases

7. Is there round-the-clock availability of PLT components (if not, how do you manage thrombocytopenia, and what measures do you take in case of unavailability?

• Yes

• No ______________________

8. Do patients pay for PLT components out-of-pocket?

• Yes

• No

9. Does your selection of the PLT component (i.e., pooled or apheresis PLTs) depend on the difficulty of the patient's solvency?

• Yes

• No

• Other ______________________

For paediatric oncology specialists

1. How long have you worked with solid tumour patients under 18 years of age? ______________________ year (s)

**2. **When do you consider thrombocytopenia?

PLT level below ______________________

3. When do you perform PLT transfusions in patients with solid tumours <18 years of age?

a) For prophylaxis, when PLT count is below

• from 5,000/mcL

• from 75,000/mcL

• from 50,000/mcL

• from 20,000/mcL

• from 10,000/mcL

• from 5,000/mcL

• Other ______________________

b) Only on-demand (in case of bleeding)

• Minor (petechiae, epistaxis) bleeding

• Heavy bleeding

• In both of the above-mentioned cases

4. When do you perform PLT transfusions in febrile patients with solid tumours <18 years of age?

a) For prophylaxis, when PLT count is below

• from 75,000/mcL

• from 50,000/mcL

• from 20,000/mcL

• from 10,000/mcL

• from 5,000/mcL

• Other ______________________

b) Only on-demand (in case of bleeding)

• Minor (petechiae, epistaxis) bleeding

• Heavy bleeding

• In both of the above-mentioned cases

5. Is there round-the-clock availability of PLT components (if not, how do you manage thrombocytopenia, and what measures do you take in case of unavailability?

• Yes

• No ______________________

6. Do patients pay for PLT components out-of-pocket?

• Yes

• No

7. Does your selection of the PLT component (i.e., pooled or apheresis PLTs) depend on the difficulty of the patient's solvency?

• Yes

• No

• Other ______________________

For paediatric oncology/haematology residents

1. How long have you worked with blood disorders among patients under 18 years of age? ______________________ year (s)

2. When do you consider thrombocytopenia?

PLT level below ______________________

**3. **When do you perform PLT transfusions in patients <18 years of age with haematologic malignancies (except APL) ?

a) For prophylaxis, when PLT count is below

• from 75,000/mcL

• from 50,000/mcL

• from 20,000/mcL

• from 10,000/mcL

• from 5,000/mcL

• Other ______________________

b) Only on-demand (in case of bleeding)

• Minor (petechiae, epistaxis) bleeding

• Heavy bleeding

• In both of the above-mentioned cases

4. When do you perform PLT transfusions in patients <18 years of age with APL?

a) For prophylaxis, when PLT count is below

• from 75,000/mcL

• from 50,000/mcL

• from 20,000/mcL

• from 10,000/mcL

• from 5,000/mcL

• Other ______________________

b) Only on-demand (in case of bleeding)

• Minor (petechiae, epistaxis) bleeding

• Heavy bleeding

• In both of the above-mentioned cases

5. When do you perform PLT transfusions in patients with solid tumours <18 years of age?

a) For prophylaxis, when PLT count is below

• from 75,000/mcL

• from 50,000/mcL

• from 20,000/mcL

• from 10,000/mcL

• from 5,000/mcL

• Other ______________________

a) Only on-demand (in case of bleeding)

• Minor (petechiae, epistaxis) bleeding

• Heavy bleeding

• In both of the above-mentioned cases

6. When do you perform PLT transfusions in febrile patients <18 years of age with haematologic malignancies (except APL) ?

a) For prophylaxis, when PLT count is below

• from 75,000/mcL

• from 50,000/mcL

• from 20,000/mcL

• from 10,000/mcL

• from 5,000/mcL

• Other ______________________

b) Only on-demand (in case of bleeding)

• Minor (petechiae, epistaxis) bleeding

• Heavy bleeding

• In both of the above-mentioned cases

7. When do you perform PLT transfusions in febrile patients <18 years of age with APL?

a) For prophylaxis, when PLT count is below

• from 75,000/mcL

• from 50,000/mcL

• from 20,000/mcL

• from 10,000/mcL

• from 5,000/mcL

• Other ______________________

b) Only on-demand (in case of bleeding)

• Minor (petechiae, epistaxis) bleeding

• Heavy bleeding

• In both of the above-mentioned cases

8. When do you perform PLT transfusions in febrile patients with solid tumours <18 years of age?

a) For prophylaxis, when PLT count is below

• from 75,000/mcL

• from 50,000/mcL

• from 20,000/mcL

• from 10,000/mcL

• from 5,000/mcL

• Other ______________________

b) Only on-demand (in case of bleeding)

• Minor (petechiae, epistaxis) bleeding

• Heavy bleeding

• In both of the above-mentioned cases

9. Is there round-the-clock availability of PLT components (if not, how do you manage thrombocytopenia, and what measures do you take in case of unavailability?

• Yes

• No ______________________

10. Do patients pay for PLT components out-of-pocket?

• Yes

• No

11. Does your selection of the PLT component (i.e., pooled or apheresis PLTs) depend on the difficulty of the patient's solvency?

• Yes

• No

• Other ______________________
